# Association of Night Snacking and Screen Time on Sleep Behavior in Japanese Toddlers: A Cross-Sectional Study

**DOI:** 10.3390/children11091083

**Published:** 2024-09-04

**Authors:** Akiko Furutani, Lyie Nitta, Seiko Mochida, Naomichi Makino, Yuki Nozawa, Yu Tahara, Shigenobu Shibata

**Affiliations:** 1 Faculty of Home Economics, Aikoku Gakuen Junior College, NishiKoiwa, Edogawa-ku, Tokyo 133-0057, Japan; shibatas@hiroshima-u.ac.jp; 2 Laboratory of Physiology and Pharmacology, School of Advanced Science and Engineering, Waseda University, Kawada-cho, Shinjuku-ku, Tokyo 162-0056, Japan; lyn@tab-el.com; 3 Benesse Educational Research and Development Institute, Ochiai, Tama City, Tokyo 734-0037, Japan; smochida@mail.benesse.co.jp (S.M.); yuki.nozawa@mail.benesse.co.jp (Y.N.); 4 The National Center for University Entrance Examinations, Komaba, Meguro-ku, Tokyo 153-8501, Japan; n-makino@rd.dnc.ac.jp; 5 Graduate School of Biomedical and Health Sciences, Hiroshima University, Kasumi, Minami-ku, Hiroshima City, Hiroshima 734-0037, Japan; yutahara@hiroshima-u.ac.jp

**Keywords:** toddlers, night snacking, screen time, sleep problem, social jet lag, chrono-nutrition

## Abstract

Background/Objectives: Irregular lifestyles, such as sleep deprivation and disordered eating, disrupt the circadian clock and are linked to health issues. This study investigates the relationship among chronotypes, social jet lag, night-snacking habits, and screen time in toddlers. Methods: A survey of 6177 mothers of children aged 3–8 years was conducted in June 2022. Means comparison and multiple regression analyses were used to analyze the data. Results: Toddlers who consumed night snacks were more likely to be evening types and experienced longer social jet lag. Longer screen time during night snacking correlated with evening-type tendencies. Juice and ice cream were identified as evening-type snacks. Conclusions: We suggest that stopping snacks after dinner is crucial to prevent evening-type eating. Especially, high-sugar and high-fat night snacks like juice and ice cream may contribute to nocturnal habits and disrupt circadian rhythms in children.

## 1. Introduction

Animals, including humans, synchronize their circadian clocks to 24 h under external light and dietary stimuli. As the circadian clock is an important factor in establishing lifestyle, environmental light schedules and food-eating styles have been identified as important factors for both circadian clocks and lifestyle.

In general, the combination of morning and night types is called a chronotype. When evening types lead a morning-starting social life, their circadian clocks and real-life clocks may not match, and they may suffer from physical discomfort. This is called the “social jet lag (SJL)”. Children with longer periods of SJL have been reported to have lower grades and quality of life. The disruption of the circadian clock can exacerbate diseases, such as obesity, diabetes, and chronic kidney disease [1].

In contrast, the shorter sleep duration of Japanese children, including toddlers, is remarkable compared to international standards [2], and sleep guidelines for preschool children have also pointed out lifestyle disruptions owing to delayed sleep onset time in children [3]. According to the “Sleep Status” by the Ministry of Health, Labor, and Welfare’s “National Health and Nutrition Survey” in 2018 [4], the percentage of “those who have not had enough rest through sleep in the past month” was 22%, which is a significant increase from 2009. Of these, it is particularly noteworthy that children sleep for the least amount of time, and in an international comparison, Japan was reported to be one of the worst countries in terms of sleep length [5].

In an experiment with teenagers, a positive correlation (r = 0.37, *p* < 0.004) was observed between academic performance and the sleep regularity index, indicating that light interventions may be effective in improving sleep regularity [6]. Regarding waking time, children who wake up earlier have higher levels of physical activity than those who wake up later [6]. Other studies have shown that a short sleep duration in early childhood is associated with obesity later in life [7] and problem behaviors, as assessed using the Child Behavior Checklist [8].

In an experiment with 5-year-olds, children with irregular habits were nearly six times more likely to be unable to draw accurate triangles than those with regular habits, suggesting that inadequate sleep is associated with delayed cognitive abilities [9]. Additionally, the amount of cortisol secreted in the saliva increases with a shorter sleep duration, suggesting an increase in sympathetic nerve function. In a longitudinal study of sleep duration, blood pressure, lifestyle, and dietary habits of elementary school children, as well as an examination of the relationship between these factors, both systolic and diastolic blood pressures were higher in children who slept less than normal, suggesting that chronic sleep deprivation increases blood pressure [10]. Sleep deprivation in toddlers and other young children affects various aspects of their health and development.

A toddler’s period of reduced bedtime regularity owing to screen time and nighttime meals may lead to a further regression of bedtime, which regresses as the developmental stage progresses; the entire sleep phase, sleep–wake, may also regress, leading to a disrupted lifestyle. Consequently, sleep quality in adulthood may deteriorate and the risk of developing diseases may increase [11]. In a survey of elementary school students, those who played TV games for 2 h or more had a lower frequency of going to bed before 10 PM than those who did not play at all [12]. This indicates that one of the causes of sleep deprivation in young children is the time spent watching screen devices, such as smartphones, tablets, TVs, and computers, and that one of the causes of sleep deprivation in young children is excessive screen time. The American Academy of Pediatrics and the Australian Academy of Pediatrics also recommended 0 h of screen time for children under 2 years of age and up to 1 h for children aged 2–5 years. Blue light exposure during screen time negatively affects sleep onset time, length, and quality. A cohort study of 1864 children of various ethnicities up to 7 years of age found that increasing TV viewing history by 1 h per day was associated with 7 min per day (95% confidence interval [CI]: 4–10) of shorter sleep [13]. It was also reported that watching television in the bedroom was associated with 31 min (95% CI: 16–45) less sleep per day [13].

Evening-type toddlers eat less or no breakfast after waking up in the morning, and their daily energy requirements are weighted toward the evening. This, combined with the fact that people sleep less and do not eat breakfast, reduces their daily activities. Additionally, they stay up longer after dinner and are hungry, which leads them to eat nighttime snacks [14]. Nighttime eating increases digestive activity and energy metabolism and raises body temperature, creating a vicious cycle that worsens the rhythm of life and sleep [15].

In addition to the above factors, the risk of lifestyle-related diseases, such as hyperlipidemia, hypercholesterolemia, and diabetes, may increase owing to increased eating at night when energy consumption is low. Nocturnal eating syndrome [16], involving misaligned eating and sleeping patterns, is linked to the development of morning appetite loss, insomnia, depressed mood, and strong appetite cravings at night [17].

A cohort study of children aged 8–10 years also investigated nighttime eating and found that the total daily energy intake time was delayed [18]. Another study reported that late-night eating was associated with reduced breakfast energy intake [19].

Although there have been some studies on the associations between the eating and sleeping habits of toddlers [20], none of the findings include a correlation between screen time and night snacking. Therefore, this study aimed to clarify the association of morning-type/evening-type and social jet lag with night-snacking habits and screen time.

## 2. Participants and Methods

### 2.1. Ethical Application

This cross-sectional study was approved by the Waseda University Human Subjects Research Ethics Review Committee (No. 2021-101, Date of approval: 28 April 2022) in accordance with the guidelines of the Declaration of Helsinki and was designed, conducted, and analyzed according to the STROBE Statement. Informed consent was obtained from all individuals who participated in the study at the start of the application use and when they responded to the web survey.

### 2.2. Participants and Questionnaires

The survey was conducted in 2022, targeting 1,331,463 monitors registered with Macromill Inc. (as of December 2022, Tokyo, Japan). For this study, an initial approach was made to 60,000 monitors who were mothers of toddlers aged 3–8 years, and a screening questionnaire was distributed. The questionnaire asked whether there were children of the target age living in the household, and responses were collected for up to the third child, allowing for multiple answers (MAs). If there were multiple eligible children, responses were randomly collected for one child. The sample size based on birth order was 23,425 for the first child, 15,770 for the second child, and 4876 for the third child.

Out of those approached, 16,890 individuals expressed their willingness to participate. From this group, recruitment was closed once the target sample size of 6000 participants was reached. Subsequently, 1030 respondents were randomly selected from each age group, with a 1:1 male-to-female ratio. Three individuals living overseas were excluded, resulting in a final total of 6177 respondents (boys: 3089; girls: 3088). Data collection was outsourced to the research firm Macromill Inc., and the survey was conducted online. Fifty questions were used to evaluate each survey item. The questionnaire took approximately 20 min to complete. All questionnaires were answered by mothers instead of the children and fathers. Four questions were asked for basic information on the sex, age, height, and body weight of the respondent’s children. Four questions were asked regarding sleep: wake-up time on weekdays with or without the help of an alarm, weekends, and holidays without a wake-up alarm, and sleep onset time on weekdays, weekends, and holidays.

Wake-up time, sleep onset, and sleep length and their respective times were investigated on weekdays, weekends, and holidays. Wake-up time, sleep onset, and sleep length are expressed in minutes. For wake-up time and sleep onset, hours were calculated as decimal numbers.

### 2.3. Evaluation Items

#### 2.3.1. Munich Chronotype Questionnaire (MCTQ) and Variables and Social Jet Lag (SJL)

The MCTQ was used to assess chronotypes from the midpoint of the free-day sleep phase. The MCTQ assesses the chronotype of individual respondents by asking about their wake-up and sleep-onset times on weekdays and unscheduled holidays. The chronotype is defined in the MCTQ as the midpoint of sleep on unscheduled holidays with correction for sleep length (MSFsc) [19]; the smaller the values of the MSFsc, the earlier the sleep onset time, which is designated as the morning-type tendency, and larger values of the MSFsc are designated as the evening-type tendency. The questionnaire used in this sleep survey utilized a Japanese version that had already been validated. Morning–evening type, SJL, screen time until bedtime, and daily screen time duration are reported in our study [21].

On weekdays, people must wake up and go to bed according to socially prescribed times to participate in social activities; however, on weekends, they may wake up and go to bed according to their circadian clocks or sleep longer to compensate for the lack of sleep during the week. This discrepancy between weekday and holiday rhythms is called “social jet lag” because it resembles the jet lag that occurs after a short flight to a country with a time difference, resulting in an increased risk of physical and mental illness and disease. SJL was calculated using the MCTQ [22], with SJL values expressed as the absolute value of the midpoint of holiday sleep hours minus the midpoint of weekday sleep hours, a method that has already been reported [23].

The MSFsc can be expressed by the following equation:

MSFsc = MSF − (SDh − SDweek)/2

MSF: Midpoint of sleep duration on unscheduled holidays.

SDh: Sleep duration on unscheduled holidays.

SDweek: Sleep duration on weekdays.

SJL can be expressed by the following equation:

SJL = Time of waking on holiday − Time of waking on weekdays.

Absolute values were calculated and analyzed as data.

#### 2.3.2. Night Snack

Definitions of late-night meals or night snacks are often not uniform. Some define a late-night meal in terms of mealtime, while others consider it a habit of eating at night regardless of the time, making it difficult to clearly distinguish between late-night and night meals.

Night snacks were defined as snacks consumed between dinner and bedtime. However, the specific times at which toddlers eat night snacks have not been investigated. Seven items were about the respondents’ children’s intake of food items during their night snacking when children ate night snacks. Toddler’s mothers could select the following multiple-choice items: juice, ice cream, vegetables, fruits, Japanese traditional sweetness, chocolate, rice crackers, cake/sweet bread, fried potato, candy/caramel, pudding/jelly, and rice bowl/noodle. As for the selection of the 12 types of night snacks, these were based on categories that ranked highly in a survey on snacking habits among young children in Japan [24]. Additionally, in a report by the Japanese Society of Public Health on a follow-up study involving 1313 toddlers aged 1.5 and 3 years, the snack items reported matched those in the above study [25]. Based on these findings, 12 types of night snacks were determined.

#### 2.3.3. Screen Time

In this experiment, screen time was defined as the time spent looking at screens, such as TV, computers, and smartphones. The definition of screen time in this study includes overall screen viewing time throughout the day (Screen time) and specifically the screen time before bedtime (Screen time before bedtime). The timing of screen time asked whether the children habitually used television, personal computers, smartphones, or tablets 1 h before sleep onset: score 1 (usually), 2 (often), 3 (occasionally), and 4 (seldom). Toddlers who showed scores 1 or 2 were combined into the “Yes” group and those who showed scores 3 and 4 were assigned to the “No” group. Screen-time durations on weekdays, weekends, and holidays were selected from 19 items (0, 5, 10, 15, 30, 60, 90, 120, 150, 180, 210, 240, 300, 360, 420, 480, 540, 600, and 720 min). Toddlers who showed 120 min or more of screen time per day were designated into the “Yes” group and those who showed 90 min or less of screen time per day were assigned to the “No” group. Children spend inordinate amounts of time on screens, televisions, cell phones, computers, and iPads, using more than one device simultaneously. For example, watching television while surfing the Internet on a tablet or mobile device; therefore, some of the screen time will be concurrent. However, this study did not investigate the presence or absence of simultaneous viewing.

#### 2.3.4. Statistical Analysis

Data are expressed as means ± SD values. All statistical analyses were performed using GraphPad Prism version 9.0.2 (GraphPad Software Inc., San Diego, CA, USA) or IBM SPSS Statistics version 28 (IBM, Japan). The D’Agostino–Pearson test was conducted for the normality of the data, and Bartlett’s test was used to examine whether the variation was equal or skewed. None of the data passed normality and/or equality; therefore, Dunn’s multiple comparisons test was applied for multi-group analysis, and the Mann–Whitney test was done for two-group analysis. * *p* < 0.05, ** *p* < 0.01, *** *p* < 0.001, and **** *p* < 0.0001.

Sample size was estimated by G power (effective size = 0.01, alpha error = 0.001, detection power = 0.95), and in the multiple regression analysis, sample size was approximately 4400 by 14 explanatory variables, including the type of night snack. Multiple regression analysis was performed using the forced entry method, adjusting for the effects of school age, sex, and type of snack in some cases as explanatory variables, and MSFsc and SJL as the objective variables.

## 3. Results

Table 1 shows the mean and SD of age, screen time before bedtime, and total screen time on weekdays and weekends for toddlers who consumed night snacks and those who did not. There were 4265 toddlers who did not eat night snacks and 1912 who ate night snacks. Their mean age was 5.50 ± 1.708 years. Those who consumed night snacks showed significantly (Mann–Whitney test) more frequent screen time before bedtime and significantly longer screen time on weekdays.

Table 2 shows the mean and SD of wake-up time, bedtime, and sleep length on weekdays and weekends, and MSFsc and SJL for toddlers with and without night snacks. The wake-up time, bedtime, and sleep length on weekdays and weekends (MSFsc) were expressed in units of clock time. SJL is expressed in hours. Bedtime was defined as sleep onset. Regardless of weekday or weekend, toddlers who ate night snacks showed significant (Mann–Whitney test) wake-up and went to bed later than toddlers who did not eat night snacks. Additionally, the sleep length of toddlers consuming night snacks tended to be significantly (Mann–Whitney test) shorter both on weekdays and weekends. Effect sizes were calculated owing to the large sample size. The effect size was particularly large for weekend sleep onset. Furthermore, toddlers who ate night snacks had slower MSFsc and longer SJL times than those who did not eat night snacks.

The results of the multiple regression analysis of the presence of night snacks, bedtime screen time scores, and total screen time on weekdays and weekends are shown in Table 3. The standardized coefficient β showed significantly higher values related to MSFsc in all four explained categories (night snack, screen time before bedtime, and weekday/weekend screen time). The standardized coefficient β tended to be highest for the presence of night snacks both in MSFsc and SJL.

**Figure 1:**
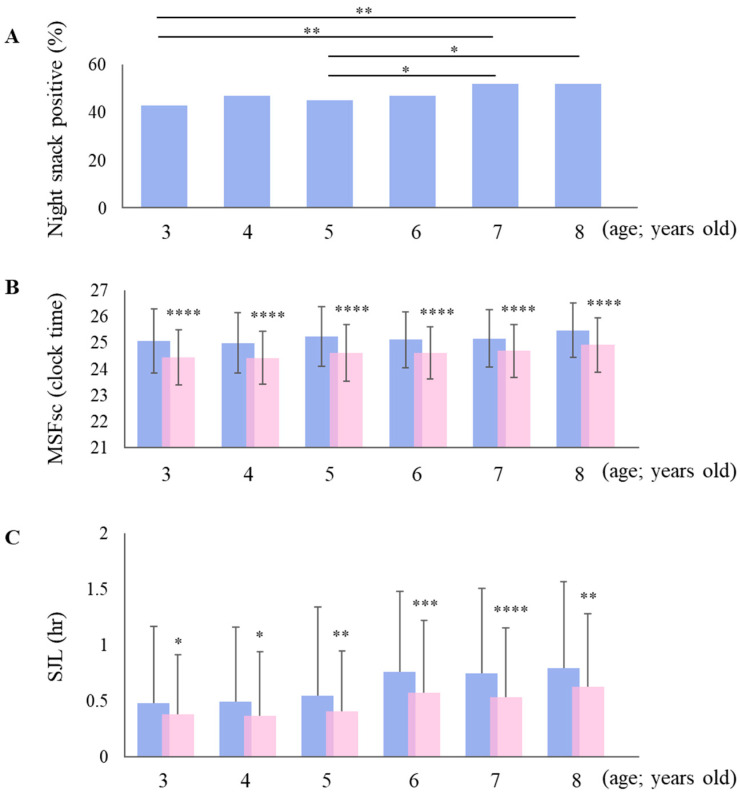
Percentage of each age group eating night snacks and the effect of their presence on MSFsc and social jet lag (SJL). Blue in the legend indicates the night snacks intake group and pink indicates the night snacks non-intake group. (**A**) is performed for toddlers of each age group vs. 7- or 8-year-old toddlers (Dunn’s multiple comparisons test, * *p* < 0.05, ** *p* < 0.01). (**B**,**C**) tests are performed on toddlers who ate night snacks vs. those who did not eat night snacks. (Mann–Whitney test, * *p* < 0.05, ** *p* < 0.01, *** *p* < 0.001, **** *p* < 0.0001).

Figure 1A shows the percentage of each age group eating night snacks, which increased predominantly with age (7–8 years) compared to younger age groups (3–6 years) (Dunn’s multiple comparisons test). The extent to which MSFsc and SJL in each age group differed by the presence or absence of night snacks is shown in Figure 1B,C. The blue in the legend indicates night snacks and pink indicates no night snacks. At all ages, toddlers consuming night snacks tended to have significantly longer MSFsc and SJL times than those not consuming night snacks (Mann–Whitney test).

Table 4 shows the results of the multiple regression analysis testing whether MSFsc and SJL interact with screen time and night snacks. The adjustment factors included age and sex. In both cases, there was no interaction. In other words, the results suggest that chronotypes, night snacks, and ST are independent of each other.

**Figure 2:**
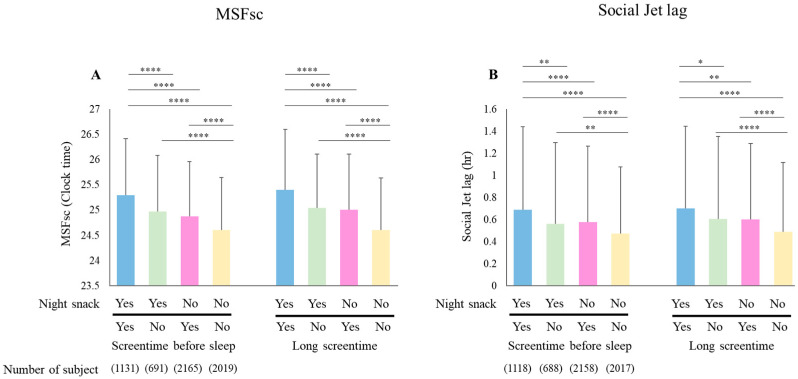
Effects of the presence of night snacking and screen time on MSFsc and social jet lag (SJL). Blue bars in the legend indicate groups with habitual night snacking and screen time problems, green bars indicate groups with habitual night snacking but no screen time problems, pink bars indicate groups with no habitual night snacking but screen time problems, and yellow bars indicate groups with neither habitual night snacking nor screen time problems. (**A**) indicates the presence/absence of screen time one hour before bedtime on the left side; of the bisection of the total daily screen time, the group with more time is shown on the right side as Yes. (**A**) indicates MSFsc hours and (**B**) social jet lag time. (Dunn’s multiple comparison test, * *p* < 0.05, ** *p* < 0.01, and **** *p* < 0.0001). The number of participants ranged from 691 to 2019.

Figure 2A compares the mean MSFsc of toddlers categorized into two groups: night snacks, screen time before bedtime, and prolonged screen time. The results show that the group with “Yes” to night snacks and “Yes” to screen time before bedtime and the group with “Yes” to night snacks and “Yes” to long screen time had higher values of MSFsc. Conversely, the group with “No” to night snacks and “No” to screen time before bedtime and the group with “No” to night snacks and little screen time (“No”) had predominantly lower values of MSFsc. Similarly, when we examined the SJL in Figure 2B, we found a similar tendency in the mean MSFsc values (Dunn’s multiple comparisons test).

Table 5 shows the results of multiple regression analysis on each of the night snacks with MSFsc and SJL as the objective variables; R^2^ indicates the contribution ratio and β the standardized partial regression coefficient. Significant differences in the factors related to MSFsc were found between the juice and ice cream. Additionally, the standardized coefficient β for SJL tended to be most positively correlated with the juice and ice cream.

## 4. Discussion

The association of the MSFsc and SJL with night snacks and screen time in toddlers was investigated. The results show that toddlers with a night snacking habit tended to wake up later, go to bed later, and have shorter sleep durations than those who did not eat night snacks. Multiple regression analysis adjusted for school age and sex identified significant differences related to MSFsc values in all four categories (night snacks, screen time before bedtime, screen time length on weekdays, and screen time on weekends). The presence of night snacks showed a notable positive correlation with MSFsc, suggesting a potential impact of night snacking on chronotypes. In an experiment involving 5254 children between the ages of 6 and 17 years, those with short sleep durations reported higher energy and macronutrient intakes at the snacking time than those with moderate sleep durations. In particular, children with extremely short sleep duration were more likely to choose sugar-sweetened beverages as snacks (16.5%) and consume them more frequently [26]. In our previous study, snacking fruit granola snacks after dinner caused a rapid increase in postprandial blood glucose levels. We found that the number of awakenings increased during sleep and showed poor-quality sleep, as assessed by the questionnaire [27]. In the current study, in particular, the standardized coefficient β suggests a positive correlation between juice and ice cream intake and MSFsc. The high sugar content in juice and ice cream may cause a reduction in sleep length and poor sleep quality; consequently, juice and ice cream intake at night will be related to late sleep onset and evening type. On the other hand, we do not know whether the screen time length and timing difference is associated with ice cream and juice intake. In future research, we should clarify the mechanism why only those two types of snacks were chosen for this study and not others.

In the present study, there was a strong relationship between nighttime snacks and screen time before bedtime. Light exposure at night causes the phase delay of circadian rhythm [28]. Thus, the delay of the circadian rhythm by night snacks and screen time until bedtime provided large MSFsc values. The association between prolonged screen time and obesity among young children, as observed by Chang et al., reinforces the need to address screen time as a modifiable risk factor in our study population. This aligns with our findings that screen time before bedtime contributes to increased MSFsc values and SJL, suggesting a broader impact of screen exposure on children’s overall health, including weight outcomes [29].

To clarify the interaction of night snacking and screen time on MSFsc and SJL, we analyzed the interaction factor using multiple regression analysis (Table 4). The results demonstrate no interaction between night snacking and screen time length or screen time before sleep, suggesting that night snacking and ST are independently associated with MSFsc and SJL. Therefore, avoiding both night snacking and ST is important for maintaining a morning-type tendency and small SJL. Furthermore, the duration of night-snacking habit should be considered, as longer-term habits may lead to more pronounced disruptions in sleep patterns and overall health. Research suggests that extended periods of high-energy, low-nutrient snacking can further impair metabolic responses. This underscores the importance of early intervention to modify prolonged snacking behaviors, aiming to improve sleep hygiene and health outcomes in children [30].

Studies examining the effects of consuming large and small amounts of snacks at night on glucose metabolism have also found that a high volume of nighttime snacks impairs the glucose response to breakfast the following day [31]. An appropriate glucose response during breakfast is a necessary component of the circadian clock reset mechanism. This is because of the mechanism by which glucose is taken up by the body and insulin is secreted. Therefore, we believe that this may be related to the fact that toddlers who eat a large number of night snacks tend to eat more evening snacks. Although traditional Japanese sweeteners are foods with a relatively high sugar content, they originally served as small sweeteners for drinking tea. Therefore, it is likely that the total amount of sugar was less than that in ice cream or juice.

In a study comparing the eating behavior of 93 adolescents after five nights each of short (6.5 h) and healthy (9.5 h) sleep, the short sleep period was associated with more carbohydrates (*p* = 0.031) and added sugar (*p* = 0.047), foods with a high glycemic load (*p* = 0.013), sweet drinks (*p* = 0.023), and low fruit/vegetable intake (*p* = 0.006) compared to the healthy sleep period. Differences in energy, fat, and carbohydrate consumption were particularly observed after 9 PM [32]. The results of this study suggest that snacks containing fat and/or sugar are preferred over nighttime snacks. We also believe that dependence on sugar content is another factor in the inability to stop night snacks. High sugar content induces rapid changes in blood glucose and insulin levels, similar to the pharmacokinetics of addictive substances. Similar to drugs of abuse, glucose, and insulin signal to the mesolimbic system to alter dopamine levels. Therefore, high sugar content induces addiction-like cravings, and highly dependent foods are rich in sugar [33].

Figure 2A,B illustrate the combined effects of night snacking and screen time on MSFsc and SJL, respectively. Groups with night snacking and screen-time issues showed higher MSFsc values and increased SJL. We believe that this is also a problem with the content of the images played during the screen time.

The aggressive advertising and marketing of high-calorie foods to children have also been implicated as potential causal factors in childhood obesity epidemics. In a study analyzing 147 commercials appearing in children’s programming on U.S. broadcast stations, commercials that increased well-being were foods with high sugar content [34]. A study that examined the amount and types of foods that children were encouraged to eat during Saturday morning television program advertisements also found that 91% of food advertisements were about foods and beverages high in fat, sodium, and sugar or low in nutrients [35]. Currently, the medium used for screen time is shifting from TV to mobile devices. However, the longer the screen time, the greater the opportunity to see foods with high sugar content, which may lead to food consumption behavior. Toddlers with night snacks tended to have larger MSFsc and SJL at all ages. However, the percentage of toddlers who consumed night snacks increased with age, particularly in the 7–8 years age group. Epidemiological reports indicate that the consumption of sweet products has increased in children and adults, especially in women of reproductive age and pregnant women [36]. However, we hypothesized that one of the reasons for this may be that in childhood, foods high in sugar content are more likely to be regulated by parental supervision, and as children grow older, their parents’ voices become more difficult to reach as they become more self-aware.

The later awakening and bedtime tendencies among toddlers with night-snacking habits may have considerable implications for their overall health. The disruption of circadian rhythms during these formative years can affect neurodevelopment and cognitive functioning [37]. Leman et al. highlighted the significant impact of sleep insufficiency and irregular bedtime on children with ADHD, which underscores the broader implications of poor sleep behavior on cognitive and behavioral outcomes. Our findings regarding late sleep onset and poor sleep quality due to night snacking and screen time suggest similar risks, highlighting the need for comprehensive sleep hygiene strategies in early childhood to support optimal neurodevelopment and attention regulation [38]. 

Our study highlights the need for health professionals to integrate nutrition and sleep hygiene guidance into routine practices. The prevalence of night-snacking habits among toddlers prompts the consideration of public health initiatives. Targeted campaigns can raise awareness of the impact of nutrition on sleep in this age group. Integrating these messages into existing programs that focus on early childhood development and parental education may yield meaningful improvements in sleep hygiene.

While this study offers valuable insights into the association between nighttime snacking and altered sleep patterns in toddlers, several limitations must be acknowledged to contextualize the findings appropriately.

Firstly, the study’s sample consisted solely of toddlers, which restricts the generalizability of the results to other age groups. The developmental stage of toddlers is unique, and as such, it remains unclear whether similar patterns would be observed in older children or adolescents. This limitation highlights the need for future research to include a broader age range to determine if the associations identified in toddlers persist or change as children grow older.

Secondly, the data collection method relied on maternal reporting through questionnaires. While mothers are generally reliable sources of information regarding their children’s behaviors, there is a potential for reporting bias. For instance, mothers may underreport or overreport certain behaviors due to social desirability or memory recall issues. This bias could mask or exaggerate the true relationship between nighttime snacking and sleep patterns. Furthermore, the reliance on maternal reporting, rather than direct assessment or self-reporting by older children, could limit the accuracy and depth of the data collected.

Another significant limitation lies in the study’s focus on nighttime snacking without sufficient granularity in categorizing snack types, frequency, or portion sizes. Different types of snacks, varying in nutritional content, could have diverse effects on sleep patterns, and the frequency and amount of snacking might also play crucial roles. Future studies should aim to subdivide these factors more precisely to better understand their individual and combined impacts on sleep. In addition, consideration should be paid to the fact that prolonged nighttime snacking habits may exacerbate disturbances in sleep and overall health. Evidence suggests that the extended consumption of high-energy, low-nutrient snacks can adversely affect metabolic responses. Therefore, future research should investigate whether early interventions to curb these habits can improve sleep and health outcomes in children. Furthermore, it remains unclear whether the duration and timing of screen exposure influence the choice of certain snacks, such as ice cream or juice, indicating the need for further research.

Moreover, this study did not account for other potentially confounding variables such as physical activity levels, overall dietary habits, and socioeconomic factors, all of which could influence both snacking behaviors and sleep patterns. The exclusion of these variables limits the ability to fully interpret the complex interplay of lifestyle factors affecting sleep.

Finally, the cross-sectional design of the study inherently limits the ability to establish causality. While the findings suggest an association between nighttime snacking and sleep disturbances, they do not confirm that snacking causes changes in sleep patterns. Longitudinal studies with controlled interventions are necessary to determine whether changes in snacking behavior directly influence sleep outcomes over time. Our study offers actionable insights for both parents and caregivers. Educating toddlers about the potential repercussions of nighttime snacking on sleep is paramount. Practical strategies such as implementing consistent bedtime routines and substituting healthier snack options are recommended. In addition, eating the largest meal of the day earlier leads to a higher-quality diet suggesting the need to consider when to eat rather than what to eat [39]. It is not that certain foods are bad; however, it is important to adopt a perspective on when to eat.

## 5. Conclusions

In conclusion, our study suggests that stopping snacks after dinner is essential to prevent evening-type snacks; however, night snacks containing high sugar and lipids, such as juice and ice cream, may make children nocturnal and cause circadian rhythm disturbances in toddlers. Although causation requires further investigation, its practical implications may be important for parents.

## Figures and Tables

**Table 1:** Characteristics of screen time in children with or without of night snacks.

Night Snack	Yes Male: *n* = 913 Female: *n* = 999		No Male: *n* = 2176 Female: *n* = 2089			
	Mean	SD	Mean	SD	*p*	D
Age (5.501 ± 1.708 years old)	5.5	1.7	5.5	1.7	0.2441	0.0264
Screen time before bedtime (score 1–4) *	2.3	1.0	2.5	1.1	<0.001	
Weekday screen time (min)	88.8	69.0	83.4	66.1	0.003	
Weekend screen time (min)	130.6	107.6	124.9	101.6	0.082	

Screen time before bedtime is use of the television, personal computer, smartphone, or tablet 1 h before sleep onset. * score 1 (usually), 2 (often), 3 (occasionally), and 4 (seldom).

**Table 2:** Characteristics of sleeping factors in children with or without of night snacks.

Night Snack	Yes Male: *n* = 913 Female: *n* = 999		No Male: *n* = 2176 Female: *n* = 2089			
	Mean	SD	Mean	SD	*p*	D
Weekday wake up (clock time)	6:72	0.6	6:60	0.5	<0.001	0.0287
Weekend wake up (clock time)	7:41	1.0	7:18	0.9	<0.001	0.2414
Weekday sleep onset (clock time)	21:34	0.7	21:08	0.7	<0.001	0.3492
Weekend sleep onset (clock time)	21:67	0.9	21:32	0.8	<0.001	0.3997
Weekday sleep length (hours)	9.3	0.8	9.5	0.7	<0.001	0.2260
Weekend sleep length (hours)	9.8	1.0	9.8	0.8	<0.001	0.1232
MSFsc (clock time)	25:17	1.1	24:74	1.1	<0.001	0.3798
SJL (hours)	0.6	0.7	0.5	0.6	<0.001	0.1611

**Table 3:** Multiple regression analysis of MSFsc or SJL with/without of night snacks and screen time.

	MSFsc		SJL	
	β	*p*	β	*p*
Night snacks (Yes or No)	−0.159	0.000	−0.064	0.000
Screen time before bedtime (score 1–4) *	−0.102	0.000	−0.057	0.000
Weekday screen time (min)	0.121	0.000	0.016	0.408
Weekend screen time (min)	0.045	0.017	0.052	0.006
	R^2^	F	R^2^	F
	0.093	103	0.051	53.7

Target variable: MSFsc or SJL; adjustment factor: age, sex. R^2^ indicates the contribution ratio and β the standardized partial regression coefficient. Screen time before bedtime is use of the television, personal computer, smartphone, or tablet 1 h before sleep onset. * score 1 (usually), 2 (often), 3 (occasionally), and 4 (seldom).

**Table 4:** Multiple regression analysis of MSFsc or SJL with screen time (ST) and night snacks.

	MSFsc					SJL			
	β	*p*	R^2^	F		β	*p*	R^2^	F
ST length	0.179	0.000	0.083	108.00	ST length	0.058	0.000	0.043	55.000
Night snacks	0.168	0.000	0.083	108.00	Night snacks	0.107	0.000	0.043	55.000
Interaction	0.004	0.858	0.083	108.00	Interaction	−0.015	0.496	0.043	55.000
	MSFsc					SJL			
	β	*p*	R^2^	F		β	*p*	R^2^	F
ST before bed time	−0.151	0.000	0.070	92.000	ST before bed time	−0.075	0.002	0.047	60.000
Night snacks	0.171	0.000	0.070	92.000	Night snacks	0.082	0.010	0.047	60.000
Interaction	0.014	0.719	0.070	92.000	Interaction	−0.008	0.830	0.047	60.000

Target variable: MSFsc or SJL; adjustment factor: age, sex.

**Table 5:** Multiple regression analysis of MSFsc or SJL with each type of night snacks.

	MSFsc		SJL		Number
Choice Items of Night Snacks	β	*p*	β	*p*	N = 1912
Juice	0.073	0.000	0.053	0.000	651
Fruits	0.024	0.091	0.014	0.344	643
Vegetables	−0.007	0.579	−0.008	0.554	123
Japanese traditional sweets	0.001	0.944	−0.019	0.185	208
Ice cream	0.090	0.000	0.042	0.010	1136
Chocolate	−0.001	0.969	−0.001	0.936	644
Rice cracker	−0.002	0.906	0.002	0.919	621
Cake or sweet bread	0.015	0.294	0.020	0.183	301
Fried potato	0.013	0.406	0.030	0.060	552
Candy or caramel	0.022	0.162	−0.007	0.653	482
Pudding or jelly	−0.027	0.081	−0.009	0.577	665
Rice boll or noddle	0.004	0.762	0.004	0.771	190
	R^2^	F	R^2^	F	
	0.036	4.85	0.051	6.79	

Target variable: MSFsc or SJL; adjustment factor: age, sex. R^2^ indicates the contribution ratio and β the standardized partial regression coefficient.

## Data Availability

The datasets used and/or analyzed during the current study are available from the corresponding author on reasonable request. The data are not publicly available due to the data size or format, which makes it impractical to share publicly.
